# Supplementary material for: Effectiveness of newborn infant parasympathetic evaluation (NIPE) index for guiding intraoperative fentanyl administration in children under 2 years: a randomized controlled trial
Source: PeerJ. 2024 Oct 8;12:e18267. doi: 10.7717/peerj.18267 (PMC11468978; doi:10.7717/peerj.18267)
Supplement: Supplemental Information 5 [file peerj-12-18267-s005.pdf]

ClinicalTrials.gov PRS DRAFT Receipt (Working Version)
Last Update: 04/21/2024 23:02

ClinicalTrials.gov ID: NCT05758090

Study Identification

Unique Protocol ID: HE651243

Brief Title: Newborn Infant Parasympathetic Evaluation (NIPE) for Guiding Intraoperative Fentanyl in Children Under 2 Years

Official Title: Comparison of Newborn Infant Parasympathetic Evaluation (NIPE) Index Versus Standard Hemodynamic Response for Guiding Intraoperative Fentanyl Administration in Children Younger Than 2 Years Under General Anesthesia

Secondary IDs:

Study Status

Record Verification: April 2024

Overall Status: Completed

Study Start: March 10, 2023 [Actual]

Primary Completion: December 31, 2023 [Actual]

Study Completion: December 31, 2023 [Actual]

Sponsor/Collaborators

Sponsor: Khon Kaen University

Responsible Party: Principal Investigator
Investigator: Thepakorn Sathitkarnmanee [tsathitkarnmanee]
Official Title: Associate Professor
Affiliation: Khon Kaen University

Collaborators:

Oversight

U.S. FDA-regulated Drug: No

U.S. FDA-regulated Device: No

U.S. FDA IND/IDE: No

Human Subjects Review: Board Status: Approved
Approval Number: HE651243
Board Name: Khon Kaen University Ethics Committee in Human Research
Board Affiliation: Khon Kaen University
Phone: +66897141913
Email:
Address:

123 Mitrapap road
Ampur Muang, Khon Kaen
40002, Thailand

Data Monitoring: No

FDA Regulated Intervention: No

Study Description

Brief Summary: During general anesthesia, objective monitoring for analgesia is still lacking. The administration of opioids relies on the experience of the anesthesiologist. There are some monitors, e.g., Analgesia Nociception Index (ANI), showing that they can evaluate analgesia in adults. Recently, a Newborn Infant Parasympathetic Evaluation (NIPE) monitor was released for assessing analgesia in children with age less than 2 years. The investigators aim to assess the efficacy of NIPE as a guide for intraoperative fentanyl administration in children under 2 years.

Detailed Description: During balanced anesthesia, hypnosis can be monitored with BIS, muscle relaxation can be monitored with a train-of-four, however, analgesia lacks precise monitoring. The anesthesiologist administers narcotics according to clinical signs and experience. Analgesia Nociception Index (ANI) has been introduced as an objective monitor for analgesia in adults. Recently, a Newborn Infant Parasympathetic Evaluation (NIPE) monitor has been introduced to assess analgesia in children under 2 years, however, clinical studies regarding its clinical efficacy are scarce.

The objective of this study is to assess the efficacy of NIPE as a guide for intraoperative fentanyl administration in children under 2 years.

Conditions

Conditions: Post Operative Pain
Analgesia
Children, Only
Narcotic Use

Keywords:

Study Design

Study Type: Interventional

Primary Purpose: Treatment

Study Phase: N/A

Interventional Study Model: Parallel Assignment

Number of Arms: 2

Masking: Double (Participant, Outcomes Assessor)

Allocation: Randomized

Enrollment: 70 [Actual]

Arms and Interventions

Arms	Assigned Interventions
Experimental: Group NIPE<br>Intraoperative fentanyl administration will be guided by NIPE protocol	Procedure/Surgery: NIPE monitor<br>Procedure/Surgery: NIPE protocol. Give intraoperative fentanyl according to NIPE value. NIPE score 50-70 indicates optimal narcotic effect. NIPE score > 70 indicated overdosage of narcotic and narcotic should be withheld. NIPE score < 50 indicates inadequate narcotic and narcotic should be given.
Placebo Comparator: Group Control<br>Intraoperative fentanyl administration will be guided by clinical signs	Procedure/Surgery: Control<br>Procedure/Surgery: Standard protocol. Give intraoperative fentanyl according to vital signs.

Outcome Measures

Primary Outcome Measure:

1. Postoperative pain: Face, Legs, Activity, Cry, Consolability scale (FLACC scale)
   Assess pain using a FLACC score every 15 minutes. FLACC has a range from 0 to 10 with 0 indicates no pain while 10 indicates worst pain. NRS of 0-3 is mild, 4-6 is moderate, and 7-10 is severe pain.

   [Time Frame: During 120 minutes in PACU]

Secondary Outcome Measure:

2. Postoperative fentanyl consumption
   Fentanyl administration in PACU

   [Time Frame: During 120 minutes in PACU]
3. Postoperative sedation score
   Sedation score (0-3; higher scores mean a worse outcome) in PACU

   [Time Frame: During 120 minutes in PACU]

Eligibility

Minimum Age: 1 Months

Maximum Age: 2 Years

Sex: All

Gender Based: No

Accepts Healthy Volunteers: No

Criteria: Inclusion Criteria:

- Newborn up to 2 years old
- ASA Status 1-2
- Undergoing elective surgery of upper part of body

Exclusion Criteria:

- Premature
- Arrhythmia
- Concomitant use of regional anesthesia
- Plan for ICU admission

Contacts/Locations

Central Contact Person: Thepakorn Sathitkarnmanee, MD
Telephone: 66-81-9547622
Email: thepakorns@gmail.com

Central Contact Backup: Sirirat Tribuddharat, MD, PhD
Telephone: 66-81-6205920
Email: sirirat.tribuddharat@gmail.com

Study Officials: Sirirat Tribuddharat, MD, PhD
Study Chair
Khon Kaen University

Locations: **Thailand**
Faculty of Medicine, Khon Kaen University
Khon Kaen, Thailand, 40002
Contact: Thepakorn Sathitkarnmanee, MD +66819547622
thepakorns@gmail.com
Principal Investigator: Sirirat Tribuddharat, MD, PhD

IPDSharing

Plan to Share IPD: No

References

Citations: **[Study Results]** Weber F, Roeleveld HG, Geerts NJE, Warmenhoven AT, Schroder R, de Leeuw TG. The heart rate variability-derived Newborn Infant Parasympathetic Evaluation (NIPE) Index in pediatric surgical patients from 0 to 2 years under sevoflurane anesthesia-A prospective observational pilot study. Paediatr Anaesth. 2019 Apr;29(4):377-384. doi: 10.1111/pan.13613. Epub 2019 Mar 21. PubMed 30793426

[Study Results] Recher M, Boukhris MR, Jeanne M, Storme L, Leteurtre S, Sabourdin N, De Jonckheere J. The newborn infant parasympathetic evaluation in pediatric and neonatology: a literature review. J Clin Monit Comput. 2021 Oct;35(5):959-966. doi: 10.1007/s10877-021-00670-8. Epub 2021 Feb 15. PubMed 33590418

[Study Results] Lim BG. Nociception monitoring tools using autonomic tone changes for intraoperative analgesic guidance in pediatric patients. Anesth Pain Med (Seoul). 2019 Oct 31;14(4):380-392. doi: 10.17085/apm.2019.14.4.380. PubMed 33329766

[Study Results] Zhang K, Wang S, Wu L, Song Y, Cai M, Zhang M, Zheng J. Newborn infant parasympathetic evaluation (NIPE) as a predictor of hemodynamic response in children younger than 2 years under general anesthesia: an observational pilot study. BMC Anesthesiol. 2019 Jun 11;19(1):98. doi: 10.1186/s12871-019-0774-y. PubMed 31185928

Links:

Available IPD/Information:
